# EXAMINING THE GERIATRIC SCHOLARS PROGRAM: IMPACT ON CLINICAL PRACTICE AMONG VA PSYCHOLOGISTS

**DOI:** 10.1093/geroni/igad104.1033

**Published:** 2023-12-21

**Authors:** Rachel Rodriguez, Priyanka Mehta, Jeffrey Gregg, Josea Kramer, Ana Alfaro, Christine Gould

**Affiliations:** Durham VA Health Care System, Durham, North Carolina, United States; VA Palo Alto Health Care System, Palo Alo, California, United States; Durham VA Medical Center, Durham, North Carolina, United States; VA Greater Los Angeles GRECC, Los Angeles, California, United States; VA Palo Alto Health Care System, Palo Alto, California, United States; VA Palo Alto Health Care System, Palo Alto, California, United States

## Abstract

Since 2014, the Geriatric Scholars Program – Psychology Track (GSP-P) has offered VA post-licensure psychologists with little-to-no prior geropsychology training an opportunity to enhance their knowledge and skills to better serve rural-dwelling older Veterans. This is achieved through an intensive course on geropsychology competencies, completion of a geriatric mental health focused quality improvement project and optional advanced learning components. A survey of GSP-P alumni was conducted to assess the long-term impact of participation on clinical practice with older Veterans and overall job satisfaction. Out of 148 alumni, 40 (27%) responded to the survey. Most alumni reported working within the home based primary care setting (45%), with the others working in a range of inpatient and outpatient settings. Utilizing a retrospective post-pre design, practice change was evaluated using a measure comprised of 12 items related to clinical practices/behaviors with older Veterans (e.g., “Incorporated a caregiver/family member in the assessment of an older Veteran.”) both before and after participating in the GSP-P. A paired samples t-test, t(29) = -2.91, p = .007, demonstrated a significant difference in clinical practice pre to post engagement with GSP-P, with participants reporting more frequent age-adapted clinical practices/behaviors post GSP-P participation. Sensitivity analyses showed a moderate effect size d = -.53 (95% CI: -.91, -.144). Qualitative responses reflected alumni growth in confidence and satisfaction in working with older Veterans, as well as more utilization of age-adapted evidenced based assessment and intervention. Overall, this evaluation shows that targeted training can improve psychologists’ clinical practice with older Veterans.

